# Supplementary material for: Are carbon emissions trading and green financial instruments synergistic? -Comprehensive quantitative research based on content analysis
Source: PLoS One. 2024 Mar 7;19(3):e0298601. doi: 10.1371/journal.pone.0298601 (PMC10919601; doi:10.1371/journal.pone.0298601)
Supplement: S1 Data — (ZIP) [file pone.0298601.s001.zip › Data/地级市Z_F工作报告.zip!/╟α║ú/╬≈─■╩╨/└┤╫╘╣1⁄2╓┌║┼í╛┬φ┐╦╩2╛▌═°í┐_2009.docx]

各位代表：

我代表市人民政府向大会作工作报告，请予审议，并请市政协各位委员提出意见。

一、2008年政府工作回顾

2008年是西宁发展历程中很不平凡的一年。一年来，在省委、省政府和市委的正确领导下，全市上下认真贯彻党的十七大、省委十一次党代会及市委十二届四次全体会议精神，深入落实科学发展观，解放思想，开拓创新，锐意进取，扎实苦干，全面完成了市十四届人大四次会议确定的各项目标任务，取得了经济社会发展最快、经济运行质量最好、城市面貌变化最大、人民群众得到实惠最多的新成就。

————综合实力实现新突破。各项经济社会发展指标创历史新高，地区生产总值迈上400亿元新台阶，达到422.2亿元，比上年增长14.7%；固定资产投资达到221.3亿元，增长23.7%；工业增加值占生产总值的比重由上年的43.1%提高到45.9%；财政一般预算收入达到58.8亿元，增长20.1%，其中地方一般预算收入达到23.4亿元，增长27.3%；实现社会消费品零售总额170亿元，增长22.3%；城镇居民人均可支配收入达到11929元，农民人均纯收入达到3944元，分别增长12.2%和16.1%。

————城市面貌发生新变化。全面完成大南山生态绿色屏障一期工程建设，三年累计造林6万亩，成为生态绿化工作的典范；实施北山危岩体综合治理，搬迁住户1160户，造林绿化603亩，有效地改善了周边环境；坚持依法拆迁、阳光拆迁，完成拆迁面积300万平方米，是城市建设历史上拆迁力度最大的一年；加快海湖新区开发及旧城改造步伐，加强城镇体系建设，推进城市化进程，市区人口增加5.03万人，城镇化率达到61%，全市呈现出大开发、大拆迁、大建设的良好局面，城市面貌改善前所未有。

————文明创建再结新硕果。领导带头，省市联动，全民参与，投入资金5.97亿元，攻坚克难，创卫工作一次性通过了专家组暗访和预评，市容市貌明显改善，使不可能的事变成了现实，使广大群众真正享受到了创卫带来的成果。创卫工作充分体现了全市干部群众积极进取的精神状态，增强了凝聚力，提高了执行力。与此同时，各类创建活动扎实推进，实现了创建国家园林城市目标，荣获全国未成年人思想道德建设工作和全国文明城市创建工作先进城市称号，市民素质普遍提高，城市形象和品位有了显著提升。

————民计民生得到新改善。多渠道筹集、整合资金53.2亿元，用于改善民生，投资比上年增加18亿元。落实就业和再就业扶持措施，下岗失业人员实现再就业1.58万人；加强社会保障工作，企业退休人员养老金月均增加116元，非公经济从业人员和农民工养老保险扩面继续推进；完善最低生活保障等社会救助体系，城市低保对象月均保障标准提高15元，农村低保年均补助标准提高240元；扎实推进保障性安居工程建设，建成廉租房1350套；大力发展教育、卫生等事业，免除了城市义务教育阶段学生杂费；新农合医疗覆盖面扩大到98.8%，提前一年实现新农合标准两年翻番的目标，人民群众的幸福指数进一步提高。

————平安建设取得新进展。面对突发事件增多、各类矛盾错综复杂的社会形势，认真落实省委、省政府的工作部署，进一步健全治安防控体系，确保了社会稳定。成功组织了奥运火炬在宁传递活动，是全国唯一受到国务院表彰的省会城市。加强安全生产和食品药品监管，严格排查和整治安全隐患，杜绝了重特大事故的发生。

一年来，我们认真落实科学发展观，牢牢抓住加快发展这个主题，重点抓了以下工作：

（一）全力应对复杂形势，经济持续平稳较快增长。面对复杂多变的经济形势，对重大问题和不确定因素超前研究，努力把挑战转化为机遇，适时采取有效措施，促进经济持续平稳健康发展。针对工业运行中运输、电力、资金等问题，加强协调，推进银企、银政合作，城投公司成功发行企业债券10亿元，缓解了资金需求压力。特别是去年第四季度以来，国际金融危机迅速蔓延和扩散，企业生产经营形势急转直下，面对严峻挑战，我们迅速行动，积极应对，认真落实国家和省上出台的政策，及时出台了扶持企业稳定发展的八条措施，帮助企业降低成本、稳定生产、扩大销售、渡过难关，抓紧产业项目建设和扩能改造，促进了经济平稳较快增长。

（二）全面落实惠农措施，新农村建设稳步推进。按照"依托城市、服务城市、发展农村、富裕农民"的总体要求，扩大特色农产品生产规模，油料产量增长17%，蔬菜产量增长26%；大力发展设施农牧业，新建日光节能温室5000栋，牲畜暖棚3880栋，建成牛羊、生猪及奶源基地42个，农牧业规模化生产效益明显提高，具有高原特色的设施农牧业发展模式初步形成；加大农村基础设施建设力度，盘道水库灌区一期工程等水利骨干项目有序实施，改善灌溉面积6.8万亩，治理水土流失82平方公里，完成63个村的人畜饮水安全工程，建成农村公路360公里、村道硬化1671公里；加快推进农业产业化，龙头企业辐射带动能力显著提高。加大农民培训转移，完成农民工技术培训2.1万人次，农民转移就业33万人次，人均劳务收入增加147元。在52个贫困村实施了整村推进扶贫项目，解决了5.98万农村贫困人口的温饱问题。

（三）积极调整产业结构，特色优势产业持续发展。在工业经济运行环境趋紧的情况下，加快调整产业结构，转变经济发展方式，加强对重点企业、重点产品的运行调控，克服各种困难，确保了全市工业经济平稳较快增长。完成工业增加值194亿元，比上年增长22.9%。不断加大投入，改善工业园区基础设施条件，千吨级多晶硅、万吨电解铜箔、百万吨复合肥、电子铝箔、再生铝等一批新项目开工建设，新能源、新材料、高原特色动植物资源精深加工等新兴产业和特色优势产业正在形成，园区实现工业增加值77.9亿元，增长74%，占全市工业的比重达到40.2%，园区经济的引领作用进一步增强。加强节能减排工作，加快重点企业技术改造，淘汰落后企业和生产设备，万元工业增加值能耗同比下降5%。

（四）加快发展特色旅游业，现代服务业日益繁荣。继续打造"中国夏都"品牌，加强旅游基础设施建设，强化旅游宣传促销，保持了旅游业稳定发展，旅游业总收入达到31.3亿元。大力发展现代流通业，朝阳铁路物流中心项目顺利开工，物流商务中心、建材综合市场等项目建成并投入营运。实施了青海虫草国际交易中心、商业巷步行街改造、珠宝玉石一条街等一批重点项目。整治农贸市场66个，主要农贸批发市场、超市实现了农产品市场准入制和票据公示制，取缔31个管理混乱的市场和早夜市，解决了长期影响市容市貌的老大难问题。继续实施"万村千乡"市场工程，建成2个连锁配送中心和4个村级综合服务中心。

（五）加强城市建设和生态保护，载体功能不断完善。加快海湖新区开发建设，青海科技馆、青海大剧院、火烧沟景观、海湖湿地公园等工程建设进展顺利。"浦宁之珠"多功能观光塔、麒麟湾、青海野生动物救护繁育中心、北门城楼建成开放，建国南路、果洛路建设工程全面完成，祁连路大修整治等工程正在实施；西过境高速公路、青藏铁路西格二线、19个城市片区景观改造等项目拆迁工作稳步推进；全面完成了24个小城建项目建设，改善了城区主要街道的交通设施；大力发展城市公交事业，特别是北京市无偿捐赠了250台公交车辆，增强了城市公交的营运能力，改善了广大市民的出行条件。加大城乡造林绿化力度，实施"三北"防护林建设、天然林保护及城区河道治理等工程，完成了西塔高速、西平高速西宁段、新宁路与黄河路景观改造和南川东路等10条街道绿化建设，城市景观水平有了进一步提升；小桥积水片区排水管网改造、污水配套管网等工程顺利完成，第三污水处理厂开工建设，城市生活污水处理率达到65%；煤烟型污染治理和重点区域环境综合整治取得明显成效。

（六）大力发展社会事业，民生保障水平得到提升。加强科技推广，引导企业技术创新，促进科技成果转化，实施各类科技项目128项。整合教育资源，加大投入力度，湟川中学、市一职迁建及农村初中校舍改造等工程进展顺利。加快发展文化产业，广泛开展群众性文化活动，全民体育健身活动蓬勃开展。完成了数字电视整体转换工作，实施了30个村广播电视"村村通"工程。加快卫生事业发展，新建社区卫生服务中心6所、村卫生室459所，建成农村无害卫生厕所1.35万座。扎实做好就业和再就业工作，城镇新增就业2.44万人。加强社会保障工作，养老保险扩面9842人、失业保险扩面3387人、医疗保险扩面1.23万人，农民工工伤保险1.29万人。完善最低生活保障、医疗、教育、取暖等救助政策，对低收入群体发放了价格补贴和生活补贴，对农村低保户发放了冬季取暖补助。建设经济适用房10万平方米，改造企业危旧房10万平方米，改造农村危房1430户。

（七）积极推进体制机制创新，发展活力明显增强。进一步转变政府职能，为公众服务、为社会服务、为市场主体服务的能力不断增强。积极推进城市管理体制改革，完善城市管理、监督、考核运行机制，城区管理水平进一步提高。推进城市交通资源优化配置，出租车行业运行质量和服务水平明显改善。深化文化体制改革，文化事业单位内在活力进一步增强，文化产业发展日趋繁荣。认真落实促进非公有制经济发展的各项政策措施，努力缓解资金瓶颈等制约因素，发展环境进一步改善。加快对外开放步伐，扩大与国内外的交流和经济协作，成功举办和承办了"青洽会暨郁金香节"、 "藏毯展览会"、"清真食品博览会"等大型经贸活动，招商引资成效显著，省外到位资金达到46亿元，增长8.7%。

（八）强化民主法制和政府自身建设，安定团结局面继续巩固。坚持重大事项报告和通报制度，自觉接受市人大及其常委会的法律监督和市政协的民主监督，共办理市人大代表建议91件、市政协委员提案126件。全面落实廉政建设责任制，强化了对行政权力的监督和制约。贯彻落实党的民族宗教政策，平等、团结、互助、和谐的社会主义民族关系得到进一步巩固。扎实推进"平安西宁"建设，严厉打击"两抢一盗"违法犯罪活动，集中整治了社会治安突出问题。加大信访工作力度，妥善处理了一批涉及群众利益的热点难点问题。多方筹措资金，盘活存量资产，积极偿还政府公益性项目拖欠工程款，有效化解了政府债务，维护了政府声誉。加强安全生产和食品药品监管，全面开展食品安全专项整治，严厉查处了"三鹿奶粉"等食品安全事件和安全生产事故。

在四川汶川等地区发生地震灾害后，我们积极组织人力物力赴灾区抢险救援，广泛发动群众踊跃捐款，倾力支援了灾区抗灾救灾和重建家园。深入开展双拥共建活动，解放军、武警官兵在维护社会稳定、抗灾抢险中发挥了重要作用。不断加强工商、统计、气象、质监等工作，计划生育、妇女儿童、残疾人等事业取得新的进步。

各位代表，在努力完成全市经济社会发展目标任务的基础上，我们坚持以人为本，多渠道筹集资金，加大民生事业投入，市委、市政府向全市人民承诺的15类60项为民办实事项目和新农村建设10项实事工程全部兑现。

回顾一年来的政府工作，成绩来之不易。这些成绩的取得，是省委、省政府和市委正确领导的结果，是市人大、市政协大力支持的结果，是全市人民共同奋斗的结果。在此，我代表市人民政府，向各位人大代表、政协委员，向各民主党派、工商联、无党派人士和人民团体，向奋斗在全市各条战线的工人、农民、知识分子、干部职工以及各行各业的建设者和劳动者，向中央驻宁单位、省直各部门、人民解放军驻宁部队指战员、武警官兵和公安干警，向所有关心、支持西宁建设和发展的各界人士，致以崇高的敬意和诚挚的感谢！

在总结成绩的同时，我们也清醒地认识到，西宁在发展中还存在一些突出问题，主要是：产业结构单一，产业链条短，支撑发展的能力不强；城市基础设施薄弱，城乡居民收入水平较低；特别是受国际金融危机蔓延的影响，工业增速明显回落，经济下行压力增大，就业形势严峻，对全年经济平稳增长带来新的难度；政府部门的服务水平离人民群众的要求还有一定的差距，个别干部的工作作风还需要进一步转变。对于这些困难和问题，我们必须采取有效措施，认真加以解决。

二、2009年工作的总体要求和主要任务

2009年，是我们全面贯彻落实科学发展观，推进"十一五"规划顺利实施的关键一年，也是应对金融危机、保持经济平稳较快增长的一年。当前，国际金融危机对实体经济的影响还在进一步加深，经济发展的不确定因素增多，面对复杂多变的形势，我们务必坚定加快发展的信心，认清和把握发展中蕴含的重大机遇，特别是中央出台的一系列扩大内需、促进经济增长的政策措施，以及支持青海等藏区经济社会发展的意见，即将开工建设兰新铁路第二双线，等对西宁发展带来了新的机遇；同时，西宁多年的发展积累，也为我们加快发展奠定了很好的基础。

今年的政府工作要按照市委十二届六次全体会议的总体要求，全面贯彻党的十七大、十七届三中全会和省委十一届五次全体会议精神，深入学习实践科学发展观，围绕保增长、扩内需、调结构、促消费、重民生的工作主线，加大投入力度提高发展能力，调整产业结构转变发展方式，强化城市建设增强服务功能，重视环境建设推进生态文明，加强社会建设改善民计民生，努力在应对挑战中确保经济社会平稳较快发展。

经济社会发展的主要奋斗目标是：地区生产总值增长12%；全社会固定资产投资增长25%；地方一般预算收入增长15%；社会消费品零售总额增长16%；单位生产总值能耗下降4%；城镇居民人均可支配收入和农民人均纯收入分别增长8%和9%；居民消费价格总水平涨幅控制在6%左右；城镇登记失业率控制在4.5%以内，人口自然增长率控制在8&permil;以内。

上述目标是预期性和导向性的，力图体现科学发展、和谐发展的要求，力求实现速度与质量结构效益相统一、经济与社会发展相协调、人口与资源环境相和谐。提出这样的奋斗目标是积极的，也是科学的，符合西宁现阶段的发展和现实需要。

全面完成今年的奋斗目标，必须把保增长促发展作为政府工作的首要任务，善于全面把握客观环境的发展变化，从宏观、中观、微观不同层面分析形势，抢抓机遇，牢牢把握发展的主动权，采取有效措施，确保经济平稳较快增长；必须把增加投资和扩大内需作为保增长的着力点，善于变压力为动力、化挑战为机遇，狠抓项目工作和招商引资两个关键不放松，不断扩大社会消费，把潜在的机遇转化为推动发展的现实生产力；必须把调整结构和转变发展方式作为促发展的主攻方向，善于利用资源转换战略，推进技术改造和创新，调整产业产品结构，大力发展循环经济，培育新型产业，做大支柱产业，做强特色产业，增强产业竞争优势和抵御风险能力；必须把深化改革和扩大开放作为促发展的根本动力，善于抓住影响科学发展的关键环节和突出问题，促进体制机制创新，不断增强经济社会的发展活力；必须把改善民生作为政府工作的出发点和落脚点，善于办好人民群众最直接、最关心、最现实的实事，实现好、维护好最广大人民群众的利益，共建共享发展改革成果。

全力做好以下十个方面的工作：

（一）做深做细项目工作，努力扩大投资规模

紧紧抓住中央扩大内需等机遇，以更大力度、更快速度、更高水平推进项目建设，扩大投资规模，为经济增长提供有力支撑。

精心谋划项目。认真贯彻国家宏观调控政策，把握国家资金投向的变化，加快编制和实施一批打基础、谋长远、得民心的重大基础设施项目、产出性项目和民生项目。借助外力提高项目规划、设计和论证水平，重点选择基础设施、生态建设与保护、特色优势产业、医疗卫生、教育、文化等项目，强化投资力度，扩大建设规模。同时，积极储备一批后续项目，既注重规划枢纽性、基础性、网络化的公共服务项目，又注重策划科技含量高的产业项目。

多渠道筹集项目建设资金。继续加大工作力度，抓好国债资金和各类专项资金的落实，发挥政府投资的引导作用；拓展各类融资平台，引导银行加大信贷投入；有效利用资本市场，推进企业直接融资；放宽市场准入条件，拓宽民间投资渠道和领域，为项目提供资金保障。加强项目资金监管，防止低水平重复建设，杜绝工程腐败现象，提高投资效益。

全力抓好项目落实。完善项目建成投用一批、开工建设一批、前期突破一批的梯次开发机制，抓好项目各个工作环节的落实，加快项目进度。抓紧各类项目进展情况的排查，及时解决项目建设中存在的困难和问题，确保项目顺利实施。对开工准备已经就绪的项目，帮助落实业主和资金，创造条件；对前期工作不充分的，扎实做好相关工作，力争列入国家、省上重点项目计划。

（二）扎实做好"三农"工作，加快城乡一体化进程

进一步加大对农业、新农村建设的投入和农民生产生活的补贴，切实加强农业基础地位，增加农民收入，促进农村各项事业发展。

加快发展现代农业。积极调整农业结构，加快建设优势产业带，抓好"菜篮子"工程建设，发展和提升以蔬菜、养殖为主的设施农牧业，年内新建农业标准化示范基地15万亩，新建日光节能温室5000栋、牲畜暖棚3000座；围绕发展特色农畜产品，进一步在精深加工上下功夫，重点培育技术起点高、发展潜力大、辐射带动能力强的龙头企业，提升油菜、蚕豆、马铃薯、牛羊肉、乳制品、绒毛、沙棘等产品加工档次。加强农业生产服务体系建设，集中建设一批批发市场、零售市场和冷藏保鲜库。积极引导和支持农民创办各类专业合作社，不断健全农业生产服务体系。

加强农村基础设施建设。加快盘道水库灌区等重点项目建设进度。统筹建设一批农村水、电、路、沼气、太阳能灶等项目，解决8万人、6万头（只）牲畜的饮水安全问题，建成农户沼气池11120座，新建农村公路185公里，硬化村道300公里。高标准建设村级综合服务中心，努力为农民群众提供全方位、综合式的服务。

统筹城乡经济发展。一是推进"三个集中"，做好项目布局规划，推进产业向园区集中；因地制宜建设农民新居，引导农民向城镇集中；鼓励农民通过多种形式流转土地承包经营权，发展多种形式的适度规模经营，使土地向经营大户集中。三县各选择1——3个条件较好的村，进行"三集中"试点工作。二是推进新农村建设，完成150个村的规划编制，抓好三县新农村典型村建设，力争到 "十一五"末每个乡镇建成3——5个典型村。三是加大整村推进、产业化扶贫、异地扶贫力度，强化社会帮扶、对口帮扶、村企共建等措施，增强贫困地区和贫困群众的自我发展能力，解决54个村、4.5万农村贫困人口的温饱问题。

大力发展劳务经济。坚持就近就地转移劳动力，扩大农村劳动力转移就业规模。积极探索城乡劳动力一体化培训的新路子，全面实施"阳光工程"、"农牧业科技培训工程"、"雨露计划"，加强务工农民技能培训，推动技能型农村劳动力稳定输出。年内培训农村劳动力1.5万人，转移就业30万人次，其中新增长期稳定就业2万人。

（三）调整优化经济结构，提升特色产业发展层次

坚持不懈地推进转变经济发展方式，大力调整产业结构，加快工业园区发展，推进企业自主创新，促进特色工业发展，不断增强工业竞争优势和抵御风险的能力。

着力提升特色产业层次。一是按照产业集中、产品延伸、资源综合利用的要求，通过调整优化产业结构，采取有效措施推动产业链向下延伸，鼓励生产制造企业从单纯的加工环节向技术创新、产品研发等环节延伸，培育新型产业，形成一批新的主导产品，提升产业竞争力。二是抓好重点项目的组织实施，新能源产业要大力发展太阳能、风能发电、动力锂电池等项目；新材料产业要重点发展铜箔、铝箔、轻金属合金材料等项目；装备制造业要提升改造传统数控机床、环卫设备、石油机械等行业；高原特色动植物精深加工产业要大力发展中藏药、生物制品和农副产品精深加工业；民族特色产业要推进藏毯、民族服饰等扩大产业规模。三是不断完善西宁经济技术开发区基础设施建设，拓展园区发展空间，为大项目落地创造条件；加快建设三县工业园区，发展协作配套的特色中小企业，发展矿产资源开发利用、农畜产品加工等产业。

推动企业自主创新和技术进步。引导和支持企业把技术改造同结构调整、产业升级结合起来，增加技术创新投入，提升行业整体水平和产业竞争力。依托骨干企业，突出新材料、新能源等产业的自主创新，集中力量研发和突破关键技术，努力培育市场前景好、产业关联度大的拳头产品。鼓励企业充分利用资本市场，通过发行债券、企业上市等方式，开展直接融资，多渠道引进资本和项目，实施资产重组，培育一批主业突出、创新能力强、带动作用大、具有较强竞争力的企业集团。

全力支持企业健康发展。认真落实扶持企业发展的政策措施，确保重点行业、重点企业正常生产。引导金融机构增加信贷规模，加大对企业尤其是中小企业的贷款支持。适时调整产品结构，抓紧技术改造和扩能升级，通过采用新技术、新工艺、新设备、新材料，增强市场应变能力和竞争能力

（四）突出旅游业龙头作用，推进服务业再上新水平

坚持以发展特色旅游业为龙头，努力打造高原旅游目的地，培育特色旅游品牌，塑造"中国夏都"形象，带动服务业发展，增强消费拉动能力，提高服务业对经济增长的贡献率。

着力提升旅游业档次。继续围绕建设高原旅游名省的目标，打造环西宁"中国夏都"旅游圈。加快旅游资源的开发、整合和优化，加强重点景区建设，不断完善和丰富塔尔寺、丹噶尔古城等旅游景点的基础设施和历史文化内涵。大力发展生态旅游、健康旅游、商务旅游和乡村旅游，满足不同群体的旅游需求。全力抓好旅游商品市场建设，建成昆仑玉加工园区和珠宝玉石一条街，加快青海虫草国际交易中心建设，支持发展一批具有地方资源优势和民族特色的旅游商品。推动旅游企业联合重组，集中打造1——2家有较强竞争力的龙头企业。大力开发会展资源，吸引全国各地、各行业来西宁举办会议，形成会议经济辐射带动效应。力争旅游业总收入增长20%以上。

不断提高服务业水平。认真落实有关财税、信贷、土地等政策，大力发展生产性服务业，重点发展物流企业和物流园区，培育物流市场，加快朝阳铁路物流中心、建国物流中心、青海物产建材商贸城等项目建设。把握居民消费层次升级需求，拓展新型服务领域，提高生活性服务业水平。加快农副产品市场升级改造，抓好青藏高原农副产品集散中心建设，改造新建8个标准化农贸市场。抓好中下南关、商业巷步行街等商业街改造提升，在每个城区打造1——2个精品商业街。大力发展金融、保险、中介、信息等新兴服务业，不断完善服务体系。

积极扩大社会消费。进一步强化消费拉动作用，研究制定鼓励和促进居民消费的具体政策措施，发展和完善消费信贷，积极培育住房、汽车、旅游等新的消费热点，改善消费环境，促进消费结构升级。实施"万村千乡"市场工程，落实"家电下乡"补贴政策，推进连锁经营向农村延伸，便利农民消费。

（五）积极塑造城市特色，进一步提升城市品位

按照"城市要有特色、建筑要有个性、市民要有精神"的要求，抓好城市规划的调整和完善，加快城市开发与建设，突出"城市特色"、彰显"建筑个性"，实现城市建设与民族、文化元素的融合，不断完善城市载体功能。

进一步提升城市规划水平。加强对西宁城市特色、历史文化传统等方面的研究，高起点、高标准对《城市总体规划》进行修编。充分发挥规划的宏观指导作用，引导城市科学发展。编制好区域综合交通规划、给排水专项规划及城区内控制性详细规划，进一步提高规划的前瞻性、指导性、科学性和严肃性。注重挖掘和弘扬西宁城市文化内涵，做好城市标志性建筑的规划、设计、选址等工作，使每个区都有几个独具特色的标志性建筑，彰显西宁城市建筑设计的唯一性。

加快城市建设步伐。集中精力抓好基础设施项目建设。一是围绕城市重点基础设施建设和旧城改造项目的实施，进一步加大拆迁工作力度，年内完成拆迁面积100万平方米，为城市开发建设创造基础条件。二是切实加快海湖新区开发建设，在抓紧续建工程建设进度的基础上，开工建设海湖体育中心主体育场、海湖中心广场、夏都会议中心、通海桥以及湟水北路、文亭路等重点工程。三是继续完善城市综合交通体系建设，抓好祁连路、南山路、五四西路等市区道路整修工程，增强城市主干道与次干路及支路间的连通性。配合省上做好西过境高速公路、青藏铁路西格二线、兰新铁路第二双线、西宁机场二期扩建等工程建设。四是加强重点基础设施改造，实施北山危岩体综合治理工程，抓好大十字地下商业街人防工程、火车站及广场改造、主要街道电力线入地等工程建设。加快19个城市片区景观改造步伐，确保年内取得实质性进展。重点做好周家泉、建国南路等片区改造，打造富有特色的宜居小区。五是继续抓好纺织南路、朝阳一巷等40条小街小巷改造，不断改善市民出行条件。做好西关大街、南北大街、小桥大街、建国路、胜利路综合整治，加快城市主要建筑楼体粉饰和亮化，实施重点片区"平改坡"工程，不断提升城市品位。

扎实推进城市创建工作。巩固和发展创卫成果，推进创卫攻坚，加大环卫保洁设施、背街小巷整修、旱厕改造等方面的投入，深入开展环境卫生综合整治，严格落实"门前三包"责任制，坚决防止垃圾死角反弹，千方百计解决创卫中存在的突出问题；继续加强城乡造林绿化工作，抓好城区绿化小区和街景小品建设，提高绿化建设水平；按照国家环保模范城市的标准和要求，不断改善能源结构，加大污水、垃圾、工业污染物处理力度，加强空气环境综合治理，减少污染物排放，改善城市环境质量。通过坚持不懈地努力，确保年内实现创建国家卫生城市目标，创建国家绿化模范城市和国家环保模范城市工作取得实效，以创卫等各类创建活动，促进全市文明程度大幅提升，向全国文明城市目标迈进。

切实加强城市管理。按照建设现代文明城市的要求，强化各部门的综合协调职能，整合城市资源，建立城市管理数字化网络，促进城市精细化管理。继续完善城市管理体制，制止不文明行为，创造安全、便捷、舒适的城市环境。加强交通秩序管理，加大交通执法力度，促使城市交通秩序明显转变。着眼长远，建设完善城市停车系统，抓好高容量、立体式停车场建设，缓解停车难的问题。加快公交事业发展，发挥公共服务功能，逐步实现城乡公交一体化。进一步做好城市公共基础设施管护工作，依法严厉打击破坏城市公共设施的违法犯罪行为。

加快城镇化步伐。坚持统筹规划，大力推进城镇体系建设，加强基础设施和公共服务设施建设，抓好三县县城扩容提质，加快中心镇、一般乡镇和中心村规划建设，形成经济分工有序、功能优势互补、空间布局合理、整体结构协调、具有地域和民族特色的县域城镇体系，推进城乡一体化进程。

（六）加大生态环境建设和保护，增强可持续发展能力

着眼于建设山清水秀、生态文明的高原山水花园城市，努力在"山"上下功夫，在"水"上做文章，加强环境保护与治理，促进生态保护与经济社会协调发展，使西宁的山更绿、水更清、天更蓝。

加快造林绿化步伐。启动大南山生态绿色屏障二期工程，抓好远山经济林建设，年内营造经济林1万亩，实施杨沟湾景区红叶谷生态园、九眼泉观光体验公园和塔尔山运动体验公园建设项目。认真实施"三北"防护林、天然林保护工程、沙棘基地建设等项目。切实提高造林质量，完成人工造林9.6万亩，封山育林27万亩。以城市绿廊通道建设为主线，通过城市公园、街道及广场绿化，海湖新区湿地公园、火烧沟整治、北山危岩体综合治理及景观林带建设，营造富有活力的城市绿地系统。

抓好河道水系整治。加强南川河、北川河、西川河水源地保护，实施封山育林、退耕（牧）还林（草），保护河流沿线生态湿地，涵养水源。加大市域内河道水系整治力度，提高防洪标准。实施南川河、西川河净化治理工程，改善沿岸景观。抓好北门泉、药王泉的开发与保护。加快建设第三污水处理厂、三县污水处理厂和城市供排水管网改扩建工程，新增污水处理能力10万吨，新建污水配套管网35.1公里，新建及改扩建供水管网61.6公里。加快小流域治理，治理水土流失80平方公里。

切实做好节能减排工作。加快西宁经济技术开发区循环经济试点园区实施方案的报批进度，争取尽快实施。加大国家节能减排资金争取力度，新上一批节能减排项目，加快淘汰落后生产能力，降低能耗和污染物排放，抓好城区遗留铬渣治理等项目。深入实施"蓝天、碧水、宁静"工程，加强空气质量检测，采取积极有效的空气治理措施，抓好建设工地扬尘污染、机动车尾气污染和煤烟型污染治理，年内改造燃煤锅炉250台（356蒸吨）。重点工业污染源主要污染物排放达标率达到87%以上，空气质量优良率保持在80%以上。

（七）创新体制机制，增强开放型经济发展活力

落实好国家出台的各项改革措施，运用市场化的改革取向破解经济社会发展的新问题和新矛盾，大胆探索符合科学发展观的一切改革，以体制机制创新推动经济社会发展。

继续深化各项改革。按照省政府的安排部署，做好市、县政府机构改革工作，深入推进行政管理体制改革和政府职能转变。加强对已经取消审批项目的后续监管，再取消一批行政审批项目，努力提高行政效能。加快事业单位改革步伐，建立有利于新闻事业和文化产业发展的管理体制和运行机制。健全城市管理体制改革的监督考评、巡查监管、奖惩激励和投入保障机制，完善创卫工作长效管理机制。促进财税管理体制改革，强化财政、税收收入管理，认真落实增值税转型改革，切实减轻企业税收负担。继续深化企业改革，以国家产业政策为指导，鼓励行业龙头企业、优势企业以资产、资源、品牌为纽带，加大兼并重组力度，利用有效资产稳定生产和就业。投资体制改革，健全和严格市场准入机制，完善统建制和代建制相结合的建设管理模式。深化农村改革，赋予农民更加充分而有保障的承包经营权，完善土地流转中介服务，支持农民通过多种形式发展适度规模经营。

继续提升对外开放水平。进一步优化发展环境，扩大对外宣传，推进全方位、多层次的对外交流合作，办好"青洽会暨郁金香节"、"藏毯展览会"等大型经贸活动，从整体上提高西宁开放型经济发展水平。创新招商引资方式，拓宽招商引资渠道，围绕优势产业、高新技术、生态保护建设等，加强项目推介，引进一批能够带动产业链延伸、带动循环发展、带动发展方式转变的龙头产业项目，年内招商引资到位资金增长10%以上。积极促进和扩大对外贸易，推进外贸经营主体多元化，开展多形式的国际合作，扩大高原特色农畜产品和民族用品出口，拓展国际市场。

大力发展非公有制经济。认真落实省、市非公有制经济发展大会精神，进一步完善促进非公有制经济发展的服务体系，加大政策扶持力度，加快融资担保平台建设，提高企业直接融资比重。尊重和保护非公有制企业经营者的积极性，为非公有制经济发展创造更为宽松的环境，使非公有制经济在扩大规模、做大总量上取得新突破。

（八）坚持以人为本的理念，切实提高民生保障水平

把坚持为人民幸福而发展、为社会和谐而努力、为城市生活更美好而奋斗，作为政府工作的出发点和落脚点，大力改善民生，不断提高人民群众的幸福感和满意度。

完善就业创业环境。发挥好政府投资和重大项目带动就业的作用，千方百计稳定就业，促进就业增长，城镇新增就业2.4万人。建立动态管理、动态援助机制，增加小额贷款规模，加强对城镇特困家庭成员、连续失业一年以上人员、残疾人员等困难群体的就业援助，实现城镇失业人员再就业1.2万人、"4045"人员就业2000人。继续做好高校毕业生和农村富余劳动力就业工作，支持企业增加就业岗位，尽量不裁员。进一步开发更多的公益性岗位，多安排困难群体就业，积极支持返乡农民工自主创业。加快构建政策扶持、创业服务、创业培训三位一体，平等竞争、充满活力的创业机制，以创业促进就业。

推进保障性安居工程建设。加大廉租房建设力度，将保障范围扩大到低收入住房困难家庭，结合棚户区改造、城中村改造、破产企业危旧房改造，通过集中新建、普通商品房小区配建等多种形式，年内开工建设廉租房2000套，改造企业危旧房10万平方米。改造农村危房1500户，逐步解决农村困难群众住房问题。

提高社会保障水平。不断提高城乡医保、低保、城镇养老保险标准和对低收入群体的补贴标准，逐步满足城乡不同层次群众的保障需求。探索建立与经济社会发展水平相适应的社会救助标准增长机制和应急临时救助机制，完善城乡低保对象动态管理和分类施保办法，实现应保尽保。进一步完善基本养老、医疗、失业、工伤、生育等社会保险制度。加快解决被征地农民和进城务工农民的社会保障问题，探索农村养老保险制度，不断提高农民保障水平。全面推进基本医疗保障制度建设，将在校大学生纳入城镇居民医保范围。提高城镇职工医保和新农合筹资标准与财政补助水平，适当扩大报销范围，提高报销比例。

加强食品药品安全整治和安全生产。认真贯彻《食品安全法》，深入开展食品药品安全专项整治，进一步健全食品药品安全质量标准，严格规范食品添加剂使用。强化企业质量安全主体责任，实行严格的食品安全质量追溯、召回、市场准入和退出制度。严格执行安全生产责任制和安全生产许可制度，深入开展安全生产专项整治，加大对交通、矿山、化工等行业的安全监管力度，严防重特大事故发生。

（九）大力发展各项社会事业，努力扩展基本公共服务

坚持以人的全面发展为目标，推进公共服务均等化，进一步促进各项社会事业更快发展。

继续推进科技创新。重视科普基地建设，不断加大应用技术研发和科技成果推广力度，通过技术项目引导，支持创新要素向企业聚集，不断增强各个领域的创新能力和市场竞争力。更加注重科研项目和科技领军人才的引进，完善吸引高端创新人才的机制和政策，提高科技成果转化率。

坚持教育优先发展。全面贯彻党的教育方针，着力研究实施素质教育的内容和方式，加强未成年人思想道德、文明行为的养成教育，提高教书育人的能力和水平，办好人民满意的教育。进一步增加教育投入，配置优质教育资源，集中力量抓好湟川中学、西宁世纪职业技术学校、西宁第一职业技术学校等示范学校建设。多渠道筹集资金，加快10所城区学校基础设施建设和26所农村初中校舍改造。大力发展各类职业教育，坚持职业教育统筹培训，推进东西部、城乡、校企联合办学，提高职业教育的规模和效益，特别要培养一大批农村实用人才。加快发展高中教育，缓解资源不足和择校压力。

切实保障群众健康水平。继续抓好城乡居民健康教育，搞好保健预防，倡导有利于健康的生活方式，提高全民健康水平。加强农村卫生基础设施和城市社区卫生服务体系建设，全面推进医疗卫生体制改革，为群众提供安全有效、方便快捷、价廉优质的医疗卫生服务，城市社区卫生服务人口覆盖率达到95%以上。启动建设市第一、第二人民医院综合诊疗大楼工程，建设20个城镇居民医保定点社区医疗卫生服务站。进一步提高市县（区）疾病预防控制能力，有效防止重大传染性疾病发生。认真落实人口和计划生育政策，加强对流动人口的计划生育服务和管理，稳定低生育水平。

繁荣文化体育事业。扎实推进"文化西宁"建设，抓好乡镇综合文化站、文化进村入户、文化信息资源共享工程，提高广播、电视和网络进村入户的覆盖率，完成10个乡镇文化站和30个村的广播电视"村村通"工程建设。全面启动文化产业园项目，加大非物质文化遗产和文物古迹保护开发力度，落实好文化"五个一"工程建设，打造文化精品，不断提高文化产业的效益。继续加强体育设施建设，广泛开展全民健身活动。

不断推进社区建设。继续加大投入，搞好办公用房和居民公益服务设施建设，改善社区工作环境，推进基层社会管理体制改革和创新，进一步发挥社区自治组织的功能和作用。搞好社区服务示范小区试点工作，重点开发便民服务、家政服务、物业管理服务等项目，切实提高为居民服务的水平。

继续加强统计、气象、老龄、妇女儿童、残疾人等工作，确保各项社会事业持续发展。

（十）加强精神文明和民主法制建设，为经济社会发展提供保障

以建设社会主义核心价值体系为根本，加强精神文明建设，推进民主法制进程，创新社会管理体制，提高公民思想道德素质和社会文明程度，促进社会和谐。

开展精神文明创建活动。通过开展各类创建活动，在市民中倡导学习意识、公德意识和科学、文明、健康、和谐的生活方式，强化社会公德、职业道德、家庭美德和个人品德教育，培养和塑造有理想、有道德、有文化、有纪律的文明市民，展现西宁人的精神面貌，努力提高城乡文明程度，使各族群众更加自觉地树立自信、开放、创新的青海意识，更加自觉地展示包容诚信、务实创新的西宁精神。

推进民主法制建设。加强基层民主政治建设，建立健全各项民主管理制度。继续以创建"星级达标"评比活动为载体，推进厂务公开工作逐步向民营企业延伸。不断深化村务公开工作，提高"示范点"创建水平。深入开展民主评议政风行风活动，下大气力解决损害群众利益的突出问题。认真实施"五五"普法规划，深入开展法制宣传教育和法治实践活动，提高公民的法律意识，推进各级政府依法行政。

完善社会管理机制。加强社会治安综合治理，完善社会治安防控体系，建立政府应急指挥中心，依法严厉打击各种违法犯罪活动。不断健全应急管理体制，有效应对自然灾害、事故灾难、公共卫生及社会安全等事件，提高危机管理和抗风险能力。积极推行信访代理服务制度，健全维护群众权益的各项机制，有效预防和化解各类社会矛盾，全力维护社会和谐稳定。贯彻党的民族宗教政策，加强依法管理，深入开展民族团结进步工作和"平安寺院"创建活动，维护正常的宗教秩序，巩固平等、团结、互助、和谐的社会主义民族关系。认真做好"双拥"工作，积极开展军民共建，巩固军政军民团结。

各位代表，为加快改善民生，今年我们将继续加大对农村经济发展、社会保障、教育、医疗、住房、就业和人居环境建设等方面的投入，再办一批群众热切期盼的实事好事！抓好10大类50项为民办实事项目和新农村建设10项实事工程，使广大群众实实在在感受到发展带来的实惠！

三、切实加强政府自身建设

在新的起点上，推动我市经济社会平稳较快发展，政府责任重大。我们将按照"为民、务实、清廉"的要求，坚持以人为本，进一步解放思想，转变观念，开拓创新，努力提高行政管理和服务水平，不断开创政府工作的新局面。

着力转变政府职能。创新公共服务体制，改进公共服务方式，为促进社会事业、公共事业、改善民生、维护社会和谐稳定提供更多更好的公共服务。严格落实工作责任制，将各项责任层层分解到人，做到权责分明。全面加强政府效能建设，强化绩效评估，实行政府绩效管理制度和行政问责制度，不断提高行政执行力。建立健全公务员激励约束机制和考核考评办法，提高办事效率和服务质量。

加快推进依法行政。贯彻落实《监督法》和《全面推进依法行政实施纲要》，自觉接受市人大及其常委会的法律监督和市政协的民主监督，坚持重大事项通报制度，进一步提高议案、建议和提案的办理质量。重视新闻舆论和社会监督，支持监察、审计部门依法独立监督。加强行政复议工作，完善规章制度，提高综合行政执法水平。推进政务公开，完善政府网站、新闻发布会等公开渠道，公开决策过程、执行过程和执行效果，保障人民群众的知情权、参与权、表达权、监督权。

加强勤政廉政建设。牢固树立过紧日子的思想，坚持勤俭办一切事业，严格控制公务消费，对公务购车、会议经费、公务接待费用以及政府机关出国（境）经费等支出实行零增长。认真落实廉政建设责任制，建立健全教育、监督和惩治相结合的反腐倡廉长效机制、权力运行监控机制，着力从源头上防治腐败。加大对工程建设、土地出让、产权变更、政府采购的监管，深入开展治理商业贿赂专项活动，依法治理和纠正损害群众利益的不正之风。教育和引导全体公务人员始终保持奋发向上的精神状态，廉洁从政，勤政为民，赢得人民群众的信任和支持。

切实改进工作作风。加强学习，努力提高各级领导、广大公务员实践科学发展观和驾驭市场经济的能力。坚持深入基层，加强调查研究，着力解决影响科学发展的突出问题。改进督查方式，提高督查效果，把抓落实的成效作为检验政府行政能力的重要标准。加强执政能力建设，提高服务质量与效率，把更多的精力投入到抓发展上、投入到改善民生上、投入到促进社会稳定上，树立良好的政府形象。

各位代表！机遇与挑战并存，积极应对复杂多变的国际国内形势，牢牢抓住千载难逢的历史机遇，保持经济社会平稳较快发展，是时代赋予我们的历史重任。让我们在省委、省政府和市委的正确领导下，认真学习实践科学发展观，团结带领全市各族人民，解放思想，坚定信心，开拓创新，大力弘扬"人一之，我十之"的实干精神，为建设更加繁荣、更加美丽、更加宜居的青藏高原区域性现代化中心城市而努力奋斗!
